# Do we really need a heart failure with preserved ejection fraction‐specific risk stratification strategy?

**DOI:** 10.1002/ehf2.14921

**Published:** 2024-06-26

**Authors:** Wojciech Kosmala, Thomas H. Marwick

**Affiliations:** ^1^ Institute of Heart Diseases, Faculty of Medicine Wroclaw Medical University Wroclaw Poland; ^2^ Baker Heart and Diabetes Institute Melbourne Australia; ^3^ Menzies Institute for Medical Research University of Tasmania Hobart Australia

The prevalence of heart failure with preserved ejection fraction (HFpEF) continues to grow, creating medical and economic challenges for healthcare systems. Despite recent advances in therapy, the prognosis in HFpEF is still unsatisfactory, both in terms of morbidity and life expectancy.^1^ In this clinically and pathophysiologically heterogeneous group, a means of identification of individuals at risk of adverse outcome could allow both research and practice to have the greatest impact.

Indeed, the validity of general HF scores like the MAGGIC risk score or Seattle Heart Failure Model in HFpEF patients suggests that the heterogeneity of HFpEF does not preclude the feasibility of a risk score, perhaps because of the proven predictive performance of a constellation of prognostic factors, irrespective of clinical circumstances. Nonetheless, the distinct mechanisms and clinical features of HFpEF justify the need to search for more HFpEF‐oriented prognostic algorithms that allow for effective risk stratification across the spectrum of HFpEF phenotypes.

The paper by Jia *et al*. published in the current issue of ESC Heart Failure evaluates selected multifactorial algorithms predicting outcome in HFpEF.^2^ After a literature search, the authors identified 16 studies testing 39 prognostic models for inclusion in the analysis. External validation was used to confirm the predictive performance of assessed strategies in only 10 of the 16. The most common predictors appearing in the models were patient demographics, past medical history, clinical presentation, treatments, laboratory tests, and left ventricular ejection fraction (LVEF). The C‐statistic in the validation cohorts ranged from 0.59 to 0.96 and from 0.57 to 0.79 for internal and external sets, respectively, thus indicating varying ability of these models to identify individuals at risk. The authors conclude that analytical quality of all included studies was suboptimal, resulting in an increased risk of bias. The major issues confounding the reliability of analysis were insufficient sample size, missing data and retrospective data collection. There was heterogeneity in the definition of HFpEF, which in some studies differed from the currently accepted threshold for LVEF of 50%. None of the studies included in the current paper analysis investigated the clinical applicability of tested algorithms, which heavily depends on the easiness of collection and availability of data necessary for clinical risk assessment. The authors' data indicate that we need better predictive tools in this setting.

**Figure 1:**
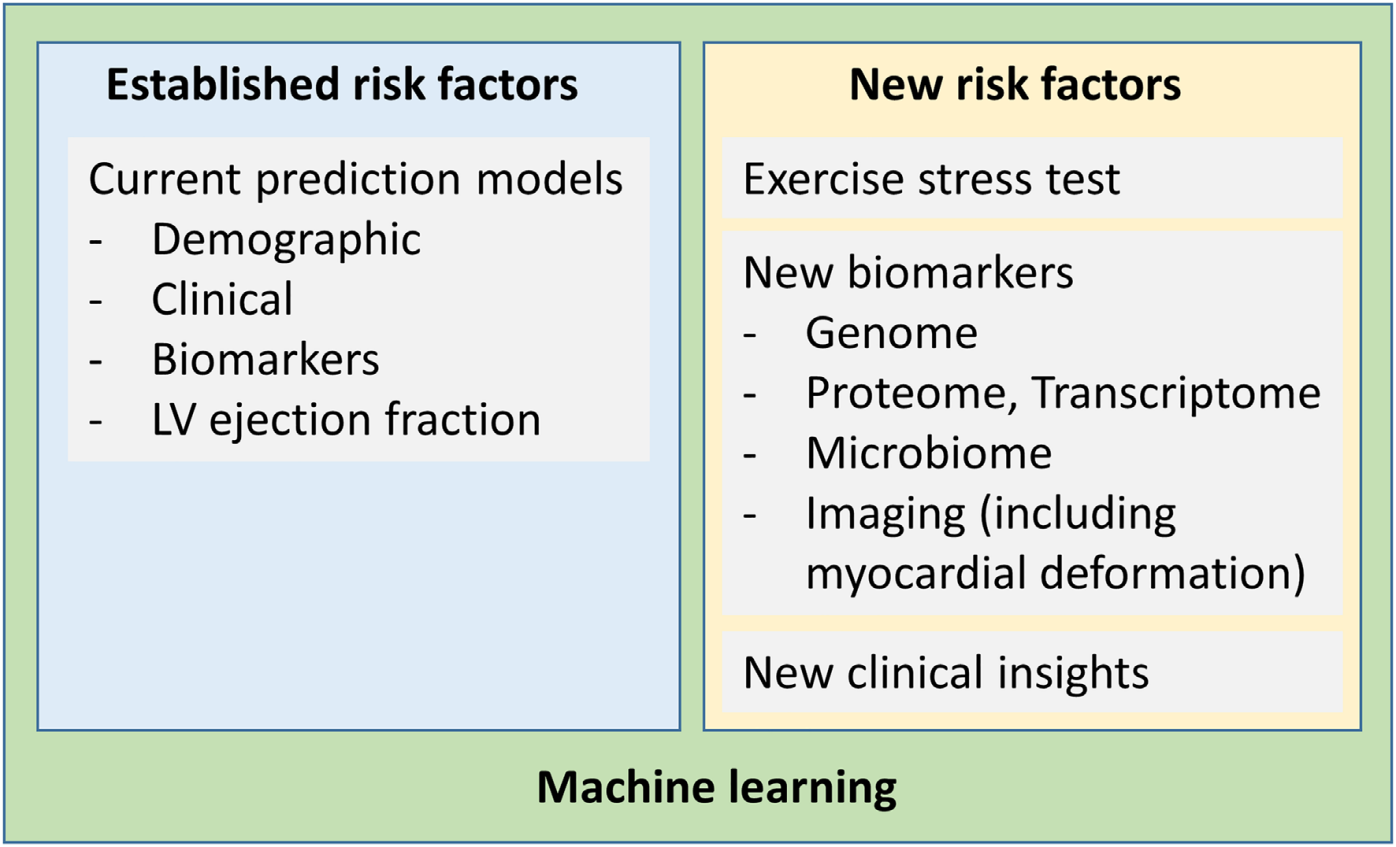
Perspective of prognostication in HFpEF.

The options for improving prediction in HFpEF include clinical, exercise, biomarker and big data approaches. First, because many components of diagnostic strategies have the potential to predict adverse outcome, such algorithms can be successfully used for risk stratification.^3^, ^4^ Second, the addition of exercise data is likely of value. When the two most commonly used scores estimating HFpEF likelihood HFAPEFF and H_2_FPEF were compared, the addition of information from the assessment at exercise provided a significant prognostic benefit over evaluation limited to resting conditions.^5^ When available, invasive haemodynamics would likely be superior. Together with findings of the prognostic significance of cardiac functional reserve,^6^, ^7^ this underscores the need for stress testing in HFpEF also for prognostic purposes. Third, HFpEF‐focused biobanks collecting HFpEF‐specific specimens and associated data might facilitate the acquisition of prognostically relevant information. Clearly, firm definition of the HFpEF phenotype, reliable input data systematically deposited in electronic medical records and accessibility of the candidate laboratory, genetic or imaging biomarkers are essential for these projects to be useful. All of these steps should inform artificial intelligence (AI) and machine learning (ML)‐based prognostic algorithms.^8^ Four studies included in the current analysis showed the superiority of these contemporary statistical approaches over traditional modelling. Indeed, accumulating evidence supports the use of AI for HFpEF prognostication. Automated hierarchical clustering can identify distinct subgroups of HFpEF patients with similar degrees of exercise intolerance but with different pathophysiological profiles, differing in terms of clinical risk.^9^ However, effective translation of findings from ML models to personalized decision‐making is not free from the application bias, the risk of which is potentiated by the non‐uniformity of HFpEF populations. Novel AI‐based approaches considering a wide spectrum of information can create a new perspective of prognostic assessment in HFpEF (*Figure* *1*).
